# Multiple Tolerization Subtractive Immunization (MTSI) Protocol: Effects on Mice and Monoclonal Antibody Specificity

**DOI:** 10.3389/fimmu.2021.760817

**Published:** 2021-12-07

**Authors:** Marina de Lima Fontes, Franciny Mara de Lima Neves, Kelvin Sousa Santos, Ana Marisa Fusco-Almeida, Maria José Soares Mendes Giannini, Sergio Luis Felisbino, Elenice Deffune, Andrei Moroz

**Affiliations:** ^1^ Department of Clinical Analysis, Monoclonal Antibody Laboratory, School of Pharmaceutical Sciences, São Paulo State University (UNESP), Araraquara, Brazil; ^2^ Department of Morphology, Extracellular Matrix Laboratory, Institute of Biosciences, São Paulo State University (UNESP), Botucatu, Brazil; ^3^ Department of Urology, Tissue Engineering Laboratory, School of Medicine, São Paulo State University (UNESP), Botucatu, Brazil

**Keywords:** monoclonal antibody, multiple tolerization subtractive immunization, cyclophosphamide, surface-epitope masking, tumor biomarkers.

## Abstract

Monoclonal antibodies (mAbs) have been a valuable tool to elucidate several biological processes, such as stem cell differentiation and cancer, and contributed to virtually all areas of biomedical sciences. Yet, it remains a challenge to obtain mAbs specific to poorly expressed epitopes, or to epitopes that are actually involved in important biological phenomena, such as cell differentiation and metastasis. Drug-induced subtractive immunization, and recently the multiple tolerization subtractive immunization (MTSI) technique, reported by our group, have the potential to level up the field, as they direct the host´s immune response towards these epitopes. However, due to cyclophosphamide (CY) treatment, high mice mortality can be observed, and only a few data are available on how these techniques affect the immune system of mice. Tolerogen and immunogen cells, RWPE-1 and PC-3 cells, respectively, were individually seeded at 2 × 10^4^ cells/cm^2^, and then adjusted to 2 × 10^6^ cells per mouse before immunization, which was conducted in a subtractive approach (MTSI) with CY. Immunosuppression of mice was recorded *via* total white blood counting, as well the reactivity of circulating polyclonal antibodies (pAbs). General parameters, including weight, physical appearance, and behavior on mice subjected to three different concentrations of CY were recorded. mAbs were obtained using classical hybridoma techniques, using the spleen of immunized mice. After purification, antibodies were characterized by Western blotting, and Indirect immunofluorescence. In conclusion, all CY dosage were efficient in creating an immunosuppression state, but only the 100 mg/kg body weight was feasible, as the others resulted in extensive mice mortality. pAbs obtained in the peripheral blood of mice showed more reactivity towards tumor cells. MAbs 2-7A50 and 2-5C11 recognized antigens from tumor cells, but not from their non-tumor counterparts, as shown in western blotting and immunofluorescence assays. MTSI technique was successful in generating mAbs that recognize tumor-specific antigens.

## Introduction

With the advent of hybridoma technology, postulated by Köhler and Milstein in 1975, and subsequent advances in biotechnology platforms, which includes phage display, mammalian cell antibody display, and transgenic animals, monoclonal antibodies (mAbs) still are prominent in the global market (1–3). Over the past three years, the number of therapeutic antibodies that entered phase I clinical studies reached 100 new molecules each year (4). By the end of 2018, 33 new antibodies for cancer treatment were in the final stage of clinical studies, being 80% of which for solid tumors (4). In a drug delivery systems era, antibody-drug conjugate (ADCs) combines affinity and specificity for a single antigenic target using mAbs coupled to a chemo/radio therapeutic agent that mediates cytotoxicity (5, 6). Moreover, clinically relevant results have been reported concerning the improvement of their pharmacological properties (e.g., increased maximum tolerated dose), which directly contributes to a decrease in the systemic adverse effects and to an increase in patient survival (6).

In addition, techniques involving radionuclide labeled antibody are extensively used in noninvasive molecular imaging techniques, such as the single photon emission computed tomography (SPECT) and the positron emission tomography (PET). These are helpful in order to detect tumor extension and residual tumor lesions that conventional imaging scans are unable to do (7). Monoclonal antibodies have also been used to identify different subsets of undifferentiated and pluripotent stem cells, their terminally differentiated phenotypes and heterogeneous phenotypes of cancer cells (8, 9). Still, there is an enormous gap that needs to be addressed regarding discovery of novel biomarkers, involved in several biological processes, for instance in the triggering and progression of solid tumors (10).

Obviously, to obtain mAbs towards novel markers one has to work with heterogeneous protein immunogens, such as whole cells, or protein cell extracts, in order to maximize the probability of recovering a mAb directed to unexplored biomarkers. Strategies that rely on pre-defined targets, although efficient for the generation of biosimilars, are of little use in discovering promising biomarkers. The problem is that, during mice immunization, these important biomarkers present in cell membranes comes together with a rich quantity of other immunogens, defined as “common” or immunodominant epitopes, which tends to elicit a stronger immune response on mice, somewhat hiding the desirable antigens to the immune system of these animals (11, 12).

The drug-induced subtractive immunization described by Matthew & Sandrock (1987) has as its goal the modulation of the humoral immune response (13). This technique uses closely related cell types derived from the same tissue: one of them called tolerogen (to which there is no desire to obtain mAbs) and another called immunogen (to which the mAbs are wished). Firstly, is induced an immune tolerance against antigenic determinants expressed in tolerogen using an alkylating agent, cyclophosphamide (CY). After tolerization, mice are immunized with the immunogen. Thus, it is expected that an effective cellular and humoral immune response directed to immunogen-specific antigens will be activated, leading to the generation of antibodies focused on “hidden” or weakly antigens, associated with virulence, immune evasion or metastatic process of malignant cells (14–16).

Cyclophosphamide (Cytoxan^®^) comprises the family of nitrogen mustards, which exhibit ability to cross-link with the DNA strand, preferentially in cells that exhibit high rate of cell duplication and renewal, such as B lymphocyte and T lymphocyte suppressor, preventing its replication (17). Although the half-life of the drug is approximately 6 hours, its side effects may extend for days or months after its administration. Weight loss, decreased appetite and taste, infertility and changes in cell composition of lymphoid organs are examples reported in studies employing different animal models (18–21).

Despite being relatively well documented in subtractive immunization techniques, to date there are no studies that reported which major side effects can be observed during its use on immunization protocol that employs multiple doses of CY. In this study, we described in details the method used to perform drug-induced subtractive immunization and the cumulative toxic effects of CY on mice using three different doses during the MTSI protocol. We also discuss the characteristics of the mAbs that were generated using this immunization technique.

## Material and Methods

### Cell Lines

The immortalized normal human prostate cell line (RWPE-1, ATCC^®^ CRL-11609) was used as tolerogen and human metastatic prostate cell line (PC-3, ATCC^®^ CRL-1435) was used as immunogen. Guidelines to maintain cultured cells were performed to according with ATCC recommendations. Cell density and viability were assessed by classic hemocytometer using trypan blue. The number of 2 × 10^6^ viable cells in 0.2 mL of phosphate buffered saline (PBS) were prepared for immunization of each mouse.

### MTSI Protocol and Physical Appearance of Mice

Twenty-four male BALB/c mice (ranging from eight to ten-weeks-old) were kept in the animal housing area at 22°C and 12 hours light/dark cycles, provided with food and water *ad libitum*. All animal manipulation respected the guidelines of animal experimentation approved by the Commission of Ethics in Animal Manipulation (CEUA/FCF/Car n° 27/2017) from the School of Pharmaceutical Sciences, UNESP. Eighteen mice were used for drug-induced MTSI protocol and another six mice were used as non-immune controls, which just received water inoculations. The immunization schedule was the following: on day 0, mice were immunized with tolerogen cells (RWPE-1, 2 × 10^6^ cells/mouse), followed by two consecutives doses of CY (24/48 hours) after immunization. This step was repeated three other times, with 15-days interval. Finally, on day 68 mice were immunized with immunogen cells (PC-3, 2 × 10^6^ cells/mouse), which was repeated four other times, with a 10-days interval between each immunization (15, 16). Three different MTSI protocols were performed: i) one with 200 mg/kg body weight (BW) of CY; ii) the second with 150 mg/kg BW; iii) another with 100 mg/kg BW. All immunizations were performed intraperitoneally.

The weight was measured on day 0, before starting the first tolerization cycle, and 10 days after its end. The same was applied for the other three tolerization cycles. Physical alterations caused by CY, including lethargy, piloerection, alopecia, lack of appetite and anorexia were also recorded, when present. To determine the standard deviation and perform the statistical analysis, the mice that died during the protocol had their weights reset to zero.

### Total Leukocyte Count

Blood from each mouse was taken from the tail vein, in heparin (Hepamax-S^®^) coated microtubes, at different times for each MTSI protocol, as follows: i) From the mice that received 200 mg/kg BW of CY, blood was taken on day 22 (three days after the second tolerization step); ii) From the mice that received 150 mg/kg BW of CY, blood was taken on day 5 (three days after the first tolerization step); And iii) From the mice that received 100 mg/kg BW of CY, blood was taken on day 39 (three days after the third tolerization step). Blood was diluted in Turk’s reagent, and the total number of leukocytes was counted and expressed in white blood cells (WBC)/mm^3^.

### Whole-Cell ELISA

To verify the reactivity of the polyclonal antibodies (pAbs) at the peripheral circulation, tail blood of the mice was taken on day 29 (ten days after the second tolerization step), and on day 63 (ten days after the fourth tolerization step), however, no anticoagulant was used. Multiwell ELISA plates (96 well) (Maxisorp, Nunc™) were coated with RWPE-1 cells (5 × 10 (4)/well) and incubated overnight at 37°C. Subsequently, the medium was removed and the wells were washed three times with PBS/Tween (0,005%), followed by incubation with blocking solution (5% w/v non-fat dry milk in PBS buffer) during 1 hour at 37°C. Polyclonal sera from each mouse (1:500) was incubated for 2 hours at 37°C, followed by secondary antibody (anti-mouse IgG/HRP, 1:5000) in the same previous condition. Finally, a developing solution was added (30 min at 37°C). Absorbance was read at 492 nm. Mouse polyclonal anti-RWPE-1 cells was used as a positive control, while mouse polyclonal anti-*Paracoccidioides* was used as a negative (isotypic) control. All samples were performed in triplicate. Cut-off values were estimated through mean absorbance obtained from the negative control multiplied by three.

### Hybridoma Production

After the immunization schedule ended, B cells from the spleen of mice immunized with 100mg/kg CY were fused with mouse myeloma SP2/0-Ag15 cells observing a ratio of 5:1 using a 50% solution of polyethylene glycol (PEG1500; Roche, Indianapolis, IN). The resulting hybridomas were seeded onto 96-well plates (Corning, Kennebunk, ME), previously coated with thymus cells from 1 week old BALB/C mice. Cells were cultured in HAT selection medium [Hybridoma-SFM (Life Technologies, Grand Island, NY), with 10% FBS (Gibco) (Life Technologies)]. At 21 days post fusion, the wells were individually screened for growing hybridoma cells. Those positive had their supernatants screened by flow cytometry or whole-cell ELISA against PC-3 cells. Positive hybridomas were cloned and as a result, mAbs 2-7A50 and 2-5C11. Mabs specificity was determined by Western blotting and Indirect immunofluorescence.

### MAbs Characterization

#### Purification of mAbs

Cell culture supernatants, containing mAbs 2-7A50 and 2-5C11, were individually harvested and purified over a Protein G affinity column (HiTrap™ Protein G Sepharose High Performance - GE Healthcare). Bound antibodies were eluted with 0.1 M glycine/HCl (pH 2.7). The obtained antibodies were immediately neutralized with 1.0 M Tris-HCl (pH 9.0), filtered in 0.45 µm mesh, and their protein content quantified using spectrophotometer at 260/280 nm.

After that, SDS-PAGE was performed using the purified antibodies 2-7A50 and 2-5C11 as samples (5 µg/lane). Stacking gel was set at 4% (running at 80V) and resolving gel at 12% (running at 100V). Additionally, a protein marker was used (BenchMark*™ Pre-stained Protein Ladder* (ThermoFischer™, 10748010). A commercially available purified murine IgG1 (Elabscience™, E-AB-F09793A) was used as control. After completion, gels were stained with Comassie Blue, overnight, and destained with 10% acetic acid/25% methanol diluted in water.

### Immunoblotting

RWPE-1 and PC-3 cell extracts were obtained by cultivating these cells, separately, in 6-well plates. Upon confluence, the monolayers were washed and a protein extraction buffer was added NP40 (Invitrogen™, with 1% proteinase inhibitor). Total protein in cell extracts were quantified using a spectrophotometer at 260/280 nm with the Pierce™ BCA Protein Assay Kit. Cell extracts (35 µg/lane) were boiled for 5 min in SDS protein sample buffer and subjected to 12% SDS PAGE at 120V for 90 min.

Proteins were transferred to a nitrocellulose membrane using the Amersham Biosciences^®^ Mighty Small Transphor at 0.4 A for 2 hours in transfer buffer (250 mM Tris and 1,92 M glicin) at pH 8.5. The blotting membranes were blocked using blocking buffer solution (5% non-fat dry milk in TBST) containing 50 mM Tris, 150 mM NaCl and 0,1% (m/v) Tween 20 for 1 h at room temperature. The purified mAbs 2-7A50 e 2-5C11 (primary antibodies) were incubated *overnight* at 4°C (1:50). The secondary antibody used was a Goat anti-mouse IgG peroxidase/HRP conjugated (Elabscience™, E-AB-1001) (1:5,000). Antibody binding was detected using the Enhanced Chemiluminescence System (ECL). Images were registered using Chemidoc XRS (Bio-Rad*™*) equipment.

### Indirect Immunofluorescence

Additionally, mAbs 2-7A50 and 2-5C11 were evaluated by indirect immunofluorescence. RWPE-1 and PC-3 cells were seeded and cultivated, individually, in coverslips. Upon reaching 60% confluence, coverslips were washed out, fixed, permeabilized with Triton X-100 (0.1%), and blocked with 2% bovine serum albumin (BSA). Nuclei were stained with Hoechst 33342 (ThermoFisher™, 62249). Selected mAbs were then incubated overnight at 4°C (1:20, at the dark) and, after washing out the coverslips, a secondary antibody was added (*Goat anti-mouse* IgG-FITC - Elabscience™, E-AB-1015) (1:100), for 1H, in room temperature. Images were acquired at 20x magnification using a fluorescence microscope (Eclipse 3000, Nikon). A positive control, anti-human CD147 (Elabscience™, E-AB-F1056A) was used at 1:100.

## Results

**Figure 1:**
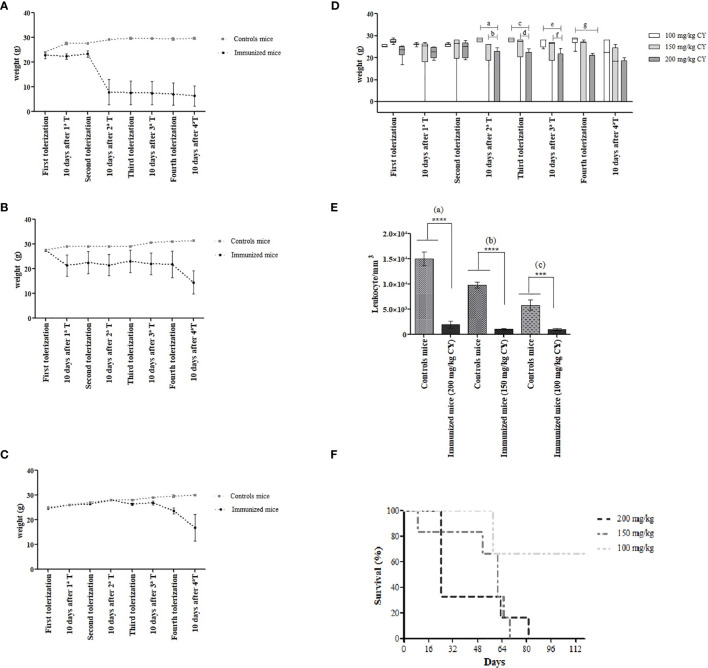
Body weight changes in BALB/c mice during MTSI protocol using **(A)** 200 mg/kg BW of CY, **(B)** 150 mg/kg BW of CY and **(C)** 100 mg/kg BW of CY. The weight was measured at the start of each tolerization cycle and ten days after the last administered dose of CY (48 hours). Box diagram showing the statistically significant differences of body weight during the immunization of mice using different concentrations of cyclophosphamide. Statistical analysis was performed using One Way ANOVA test with Sidak’s post test and multiple comparisons. ^a^p= (0.0004); ^b^p= 0.221; ^c^p= 0.0003; ^d^p= 0.0085; ^e^p= 0.0007, ^f^p= 0.0142, ^g^p= 0.0003 **(D)**. Survival analysis shows the death of 100% of mice that were treated with higher doses of CY **(E)**. Leukocyte counts/mm^3^ of the immunized mice were compared with the leukocyte counts of the non-immune mice (200 mg/kg and 150 mg/kg; p < 0.0001), (100 mg/kg; p = 0.0002) **(F)**. ****means p < 0.0001;***means p = 0.0002.

The effects caused by the administration of 200 mg/kg BW of CY, corresponding to the maximum dose prescribed for BALB/c mice, resulted in the cachectic state of all of them, with remarkable piloerection, hair loss in the dorsal region and, especially, pronounced emaciation, inactivity, evidenced by the hunched posture. These effects were more evident after the third and fourth tolerization steps, which led to the death of all immunized mice. The same was found during the second immunization schedule in which the dose of CY administered was lowered to 150 mg/kg BW of CY. Although at this dose the animals remained alive for a longer period, physical weakness was still observed with a marked decrease in weight as the cycles were repeated. The implications triggered by the administration of the drug were apparent, cumulative, and prolonged considering that most animals died shortly after the last and fourth tolerization. However, when the dose of 100 mg/kg BW of CY was administered, the mice quickly managed to recover their weight at each cycle of tolerization, not showing signs of fragility. Despite the fact that immunized mice remained alive until the fourth tolerization, two of them showed marked weight loss after the last cycle and died on day 56. The timeline of weight measurement during tolerization of BALB/c mice using the stipulated doses of CY is demonstrated in **Figure 1**.

In order to compare the different concentrations of CY and its influence on the weight in mice during the stages of the MTSI protocol, a box diagram was designed and included in **Figure 1D**. These outcomes reveal a statistically significant decrease in the weight of the immunized mice that received 200 mg/kg of CY compared to those that received 100 mg/kg, after 10 days of the second tolerization (p= 0.0004). Thus, we show that there is an indirect association between the increase in the dose of CY and the weight loss of the mice. It is important to remark that there were no statistical differences in the weight of the mice when the dose of 100 mg/kg was used compared to the dose of 150 mg/kg; however, the 150 mg/kg dosage killed all the animals during the immunization protocol. Survival analysis showed that higher doses led to the death of 100% of the immunized mice (**Figure 1E**).

During the evaluation of the immunosuppression caused by CY, it was observed that all concentrations of the drug were effective in reducing the WBC counts (**Figure 1F**). The WBC counts in the immunized mice were below the normal value (2000 to 10.000/mm^3^), indicated that CY was acting as expected, considering that lymphocytes represent 70-80% of the WBC differential count in mice (22). When the leukocyte counts of non-immune mice was compared with immunized mice into the same immunization schedule, there were statistical differences in all groups (p< 0.0001; a), (p< 0.0001; b), (p= 0.0002; c) for the concentrations of 200 mg/kg, 150 mg/kg, and 100 mg/kg of CY, respectively.

Moreover, the WBC from immunized mice showed statistical differences comparing the dose of 200 mg/kg *vs* 150 mg/kg (p= 0.0474) and 200 mg/kg *vs* 100 mg/kg (p= 0.0486). The variations observed in the number of leukocytes in the control mice may have occurred due to the stress caused during the handling and immobilization of them inside the restrain cages.

Mice serum analysis demonstrated that all tested concentrations were efficient in depleting pAbs directed to epitopes present RWPE-1 cells, as shown by the optical densities from whole-cell ELISA assay (**Table 1**). However, we have also identified that after administering the immunogen cells (PC-3), the reactivity of pAbs increase for both cell lines (data not shown). This indicates that some fast-growing B-cell clones may be producing antibodies directed towards shared epitopes, which can be sorted during the screening of hybridomas.

**Table 1:** Whole-cell ELISA optical densities of pAbs from immune and non-immune mice serum against non-tumor prostate cell.

	MTSI schedule (200 mg/kg) BW of CY		MTSI schedule (150 mg/kg) BW of CY		MTSI schedule (100 mg/kg) BW of CY	
	Absorbance (492 nm)		Absorbance (492 nm)		Absorbance (492 nm)	
Polyclonal serum	2nd tolerization	4th tolerization	2nd tolerization	4th tolerization	2nd tolerization	4th tolerization
Positive control	3.55 ± 0.08	3.55 ± 0.08	3.55 ± 0.08	3.55 ± 0.08	3.55 ± 0.08	3.55 ± 0.08
Negative control	0.17 ± 0.03	0.17 ± 0.03	0.17 ± 0.03	0.17 ± 0.03	0.17 ± 0.03	0.17 ± 0.03
Secondary control	0.06 ± 0.02	0.06 ± 0.02	0.06 ± 0.02	0.06 ± 0.02	0.06 ± 0.02	0.06 ± 0.02
Non-imune control 1	0.08 ± 0.03	0.02 ± 0.02	0.00 ± 0.00	0.00 ± 0.01	0.01 ± 0.01	0.03 ± 0.05
Non-imune control 2	0.05 ± 0.03	0.38 ± 0.03	0.03 ± 0.01	0.07 ± 0.01	0.03 ± 0.04	0.02 ± 0.02
Immunized mouse 1	*	*	0.05 ± 0.05	*	0.13 ± 0.08	0.03 ± 0.01
Immunized mouse 2	*	*	0.00 ± 0.00	*	0.03 ± 0.05	0.03 ± 0.01
Immunized mouse 3	0.12 ± 0.01	0.03 ± 0.03	0.00 ± 0.00	0.00 ± 0.00	0.03 ± 0.02	*
Immunized mouse 4	*	*	0.02 ± 0.00	*	0.03 ± 0.02	0.04 ± 0.00
Immunized mouse 5	0.04 ± 0.02	0.13 ± 0.02	*	*	0.06 ± 0.01	*
Immunized mouse 6	*	*	0.00 ± 0.00	*	0.03 ± 0.04	0.00 ± 0.00

The asterisk indicates which mice died before the sample was collected.

**Figure 2:**
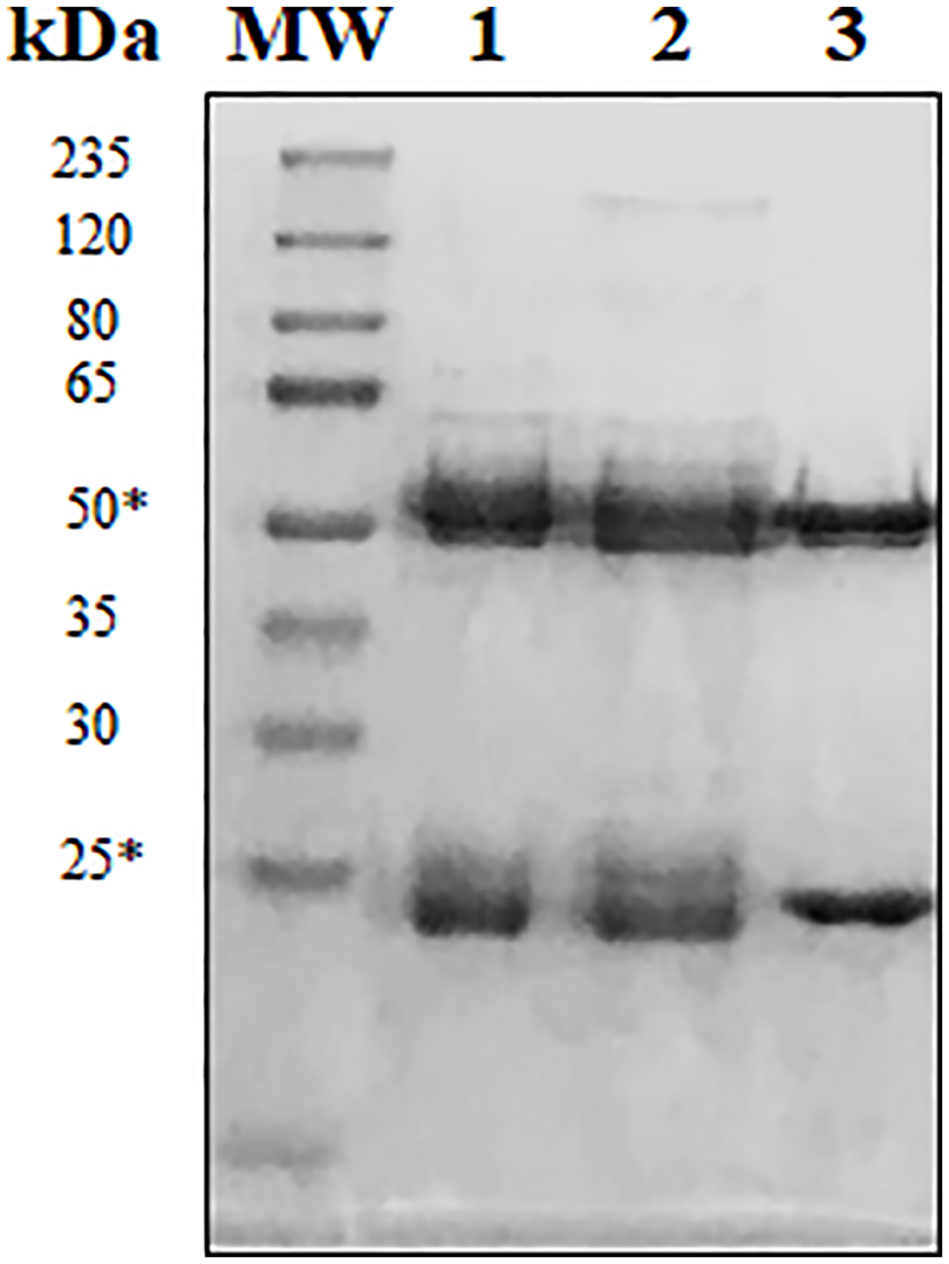
Reducing SDS-PAGE at 12% of purified mAbs 2-7A50 and 2-5C11. MW: molecular weight marker; 1: Commercially available purified murine IgG1; 2: mAb 2-7A50; 3: mAb 2-5C11. All mAbs were heated at 100°C for 5 min prior electrophoresis. Reduced samples of the IgG class from mAbs 7-A50 and 5-C11 showed heavy chains of approximately 50 kDa and light chains of approximately 25 kDa (*).

**Figure 3:**
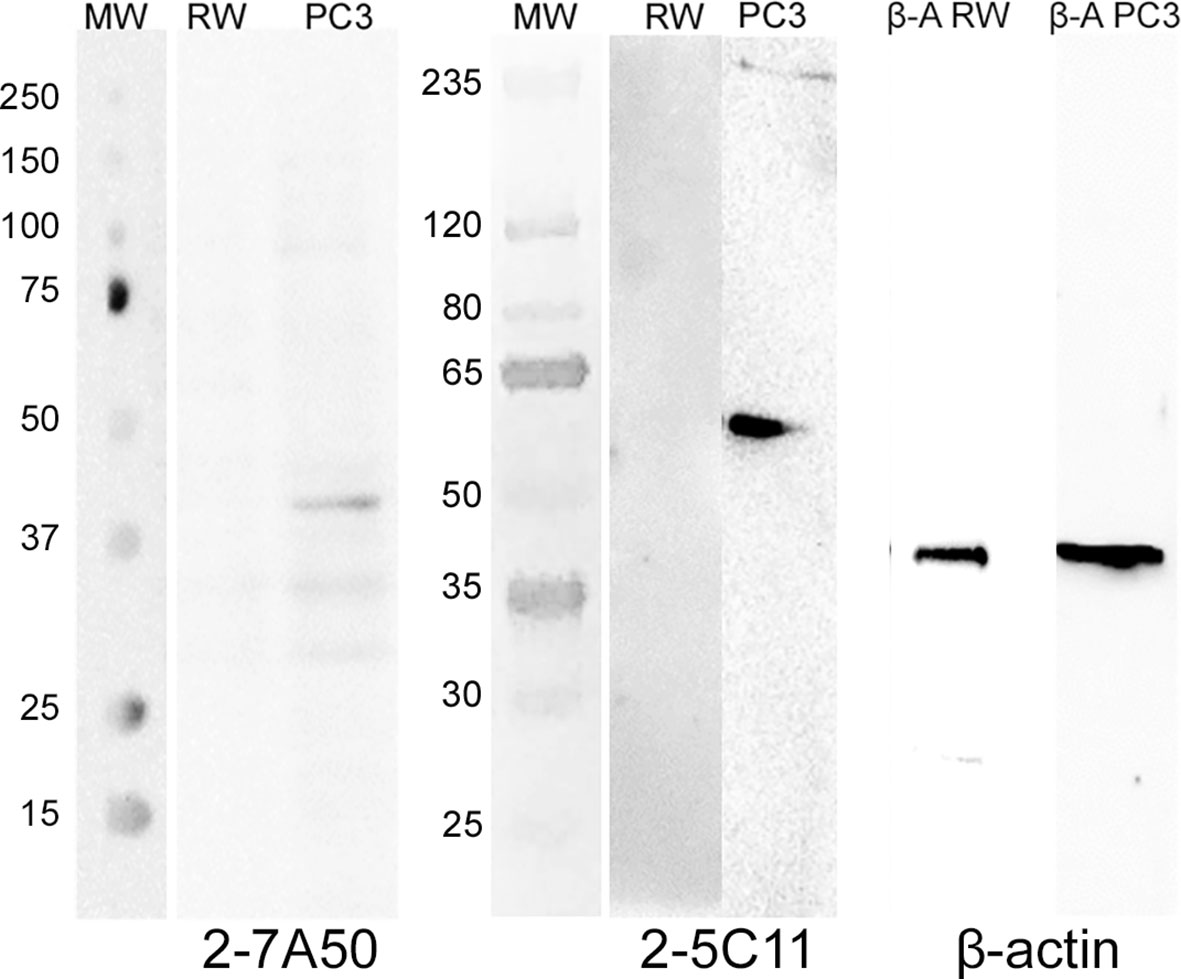
Western blotting of mAbs 2-7A50 and 2-5C11 against cell extracts from tumoral and non-tumoral cell lines. MW: molecular weight marker; RW: RWPE-1 cell line extract; PC-3: PC-3 cell line extract; β-A RW: β-actin antibody against RWPE-1 cell line extract; β-A PC-3: β-actin antibody against PC-3 cell line extract. Both mAbs recognized bands present only at the PC-3 cell line. No reactivity is shown against the RWPE-1 cell line. mAb 2-7A50 specifically detected a 45kDa band while mAb 2-5C11 specifically detected a 62 kDa band. Detection was performed using chemiluminescent method.

**Figure 4:**
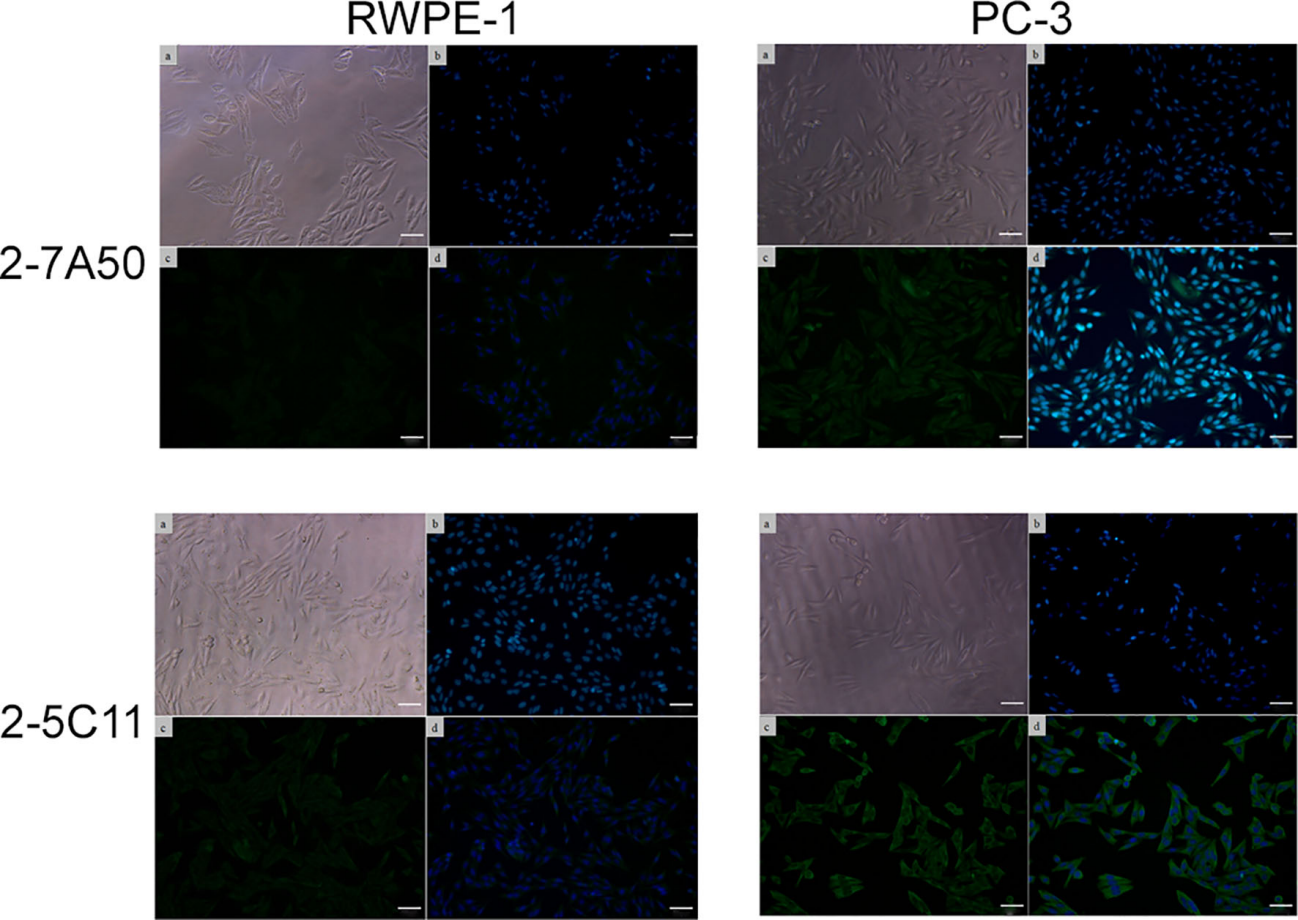
Indirect immunofluorescence of mAbs 2-7A50 and 2-5C11 against tumoral and non-tumoral cell lines. **(a)** cells under light microscopy; **(b)** nuclei stained with Hoechst 33342; **(c)** mAb staining; **(d)** mAb + nuclei staining. High reactivity is shown, for both mAbs, against the tumoral cell line. Low to none reactivity to the non-tumoral cell line is present. In both mAbs, a diffuse staining pattern is displayed.

Several hybridoma clones were successfully obtained, seven of which produced mAbs that recognized the tumoral cells differentially. Two of them were selected for further characterization (mAbs 2-7A50 and 2-5C11) and, after purification, highly visible antibody bands were seen at the SDS-PAGE method (**Figure 2**), indicating adequate concentrations (µg/µL) for further testing. Western blotting results revealed bands with approximately 45 kDa and 62 kDa, for mAbs 2-7A50 and 2-5C11, respectively. Also, there was high affinity of both mAbs for the cell extracts obtained from tumor cells (PC-3), and low to none for the cell extracts from non-tumoral cells (RWPE-1) (**Figure 3**). Moreover, this specificity towards the tumor cells was confirmed using an indirect immunofluorescence technique (**Figure 4**).

## Discussion

Recently, many technologies, including gene expression profiling, transcriptome, proteomics, and metabolomics platforms have been explored for the discovery of novel biomarkers that can reflect tumor progression, infectious, and autoimmune diseases (23, 24). Due to the heterogeneity in the tumor environment, and new mutations that cancer cells acquire during the progression from localized disease to advanced disease, identifying tumor markers is a challenge for biomedical and biotechnological research.

In this sense, researchers have been perfecting a 46-year-old technology that provides the perfect tool for identifying biomarkers: the mAb! These are reliable bioproducts, easy-to-produce, highly specific, which have been dominating the biomedical market given their high aggregated value and contribution to knowledge in general. In this technique, the immunization protocol is perhaps the most crucial event, because it directs the specificity of the obtained antibodies. It is worth to mention that if this important step is not carefully planned, one will probably end up with antibodies that won´t recognize clinically important antigens.

One of the advancements in immunization techniques, called drug-induced subtractive immunization, has demonstrated the ability to generate mAbs capable of recognizing metastatic tumor cells rather than non-tumor cells from the same tissue (25, 26). In a previous study, published by our group, we demonstrated, through flow cytometry assays, that the reactivity percentage of polyclonal antibodies (pAbs) towards non-tumor prostatic cells lowers as the tolerization steps are repeated. Two groups of immunized mice were used: i) the first that received only one tolerization step and ii) the other one that received four tolerization steps (16).

Now, to increase the performance of the described MTSI technique (16), we have now performed three different immunization schedules, using different dosages of cyclophosphamide (Cy) (200, 150 and 100 mg/kg BW). In the present study, we confirmed the extensive deleterious effects on mice that received 200 mg/kg BW and 150 mg/kg BW of CY, including progressive weight loss, hair loss, and prominent body curvature. Although these concentrations achieved the desired immunosuppression, with a marked decrease of the WBC counts and non-reactivity of pAbs to the tolerogen cells, the side effects caused by the repetition of high-doses of CY prevented the survival of the mice prior to the schedule conclusion. This is unacceptable for the production of mAbs, given that the animals must go through the entire immunization schedule. In this sense, aiming to refine this procedure, we worked with a lower concentration (100 mg/kg BW) of CY that did not demonstrate the previous undesired side effects. Mice exposed to this CY concentration maintained reasonable body weight and rapidly recovered after each tolerization step was repeated, while still decreasing WBC counts and maintaining the non-reactivity of pAbs to the tolerogen cells.

Several mAbs were obtained from hybridoma clones from B cells harvested from spleens of mice that received the 100 mg/kg CY dosage. After fusion, during screening, some of them exhibited high reactivity towards both cell lines (indicating that the epitope/antigen was shared between those cell lines), while others towards the tumoral cell line only (data not shown). This result is to be expected, given that after immunization, several B cell clones proliferate in the lymphoid organs, and only some of them will produce mAbs directed to tumor-specific antigens. Moreover, prior to cloning, several hybridoma clones will be growing together, and if the ones producing antibodies directed to shared epitopes are fast growing ones, this will make it harder to find the perfect clone. Amongst the better antibodies, mAbs 2-7A50 and 2-5C11 were selected for further investigations. As demonstrated, using different characterization techniques, both antibodies proved to be more specific to tumor-specific epitope/antigens, given that the reactivity was positive for the tumor cell line (PC-3) only. Immunofluorescence also showed a diffuse staining in membranes and cytoplasm of PC-3 cells for both antibodies. Overall, we conclude that the MTSI with CY dosage of 100 mg/kg was successful in inducing immunosuppression, and in generating mAbs specific to antigens expressed in tumor cells.

Intending to achieve even better results, we propose the association of the MTSI technique with surface-epitope masking (SEM) (which is also a subtractive immunization approach), in order to promote extra tolerance induction against immunodominant epitopes (27). SEM is a method that may be employed during the immunization step, in which the cancer cells are masked by pAbs previously obtained against non-tumor cells. These biomolecules show reactivity to both tolerogen and immunogen cells, without specificity for a single antigenic determinant. The blockage caused by the antigen-antibody binding allows only unshared/exposed epitopes in the immunogen cells to be recognized by the immune system. It is believed that, by doing so, a more effective humoral cellular response can be elicited, resulting in the generation of specific antibodies to weakly expressed antigens, associated with tumor progression/invasiveness. The combination of drug-induced MTSI with SEM is a remarkable approach that could yield results that are even more promising regarding specific monoclonal antibodies.

## Data Availability Statement

The raw data supporting the conclusions of this article will be made available by the authors, without undue reservation.

## Ethics Statement

The animal study was reviewed and approved by Commission of Ethics in Animal Manipulation (CEUA/FCF/Car n° 27/2017) from the School of Pharmaceutical Sciences, UNESP.

## Author Contributions

ML: conducted experiments, data interpretation, article draft. FN: conducted experiments, data interpretation. KS: conducted experiments, data interpretation. AF-A: data interpretation, article draft, article revision. MG: article revision. SF: article revision, partial funding. ED: article revision, partial funding. AM: data interpretation, article draft, article revision, major funding, research supervision. All authors contributed to the article and approved the submitted version.

## Funding

This article was supported by the São Paulo Research Foundation (FAPESP), grant number: 2017/20404-0 and a research funding (2018/19083-7).

## Conflict of Interest

The authors declare that the research was conducted in the absence of any commercial or financial relationships that could be construed as a potential conflict of interest.

## Publisher’s Note

All claims expressed in this article are solely those of the authors and do not necessarily represent those of their affiliated organizations, or those of the publisher, the editors and the reviewers. Any product that may be evaluated in this article, or claim that may be made by its manufacturer, is not guaranteed or endorsed by the publisher.
