# Supplementary figures and images for: Multiple Tolerization Subtractive Immunization (MTSI) Protocol: Effects on Mice and Monoclonal Antibody Specificity
Source: Front Immunol. 2021 Dec 7;12:760817. doi: 10.3389/fimmu.2021.760817 (PMC8688850; doi:10.3389/fimmu.2021.760817)

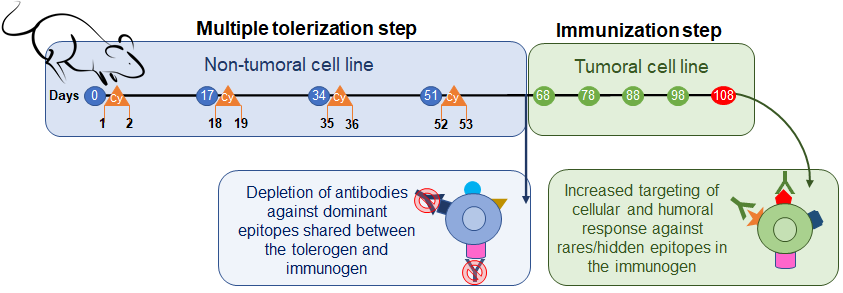

Supplement: Supplementary file 1 [file Image_1.tif]
